# Consistent expression of PD-L1 in tumor microenvironment with peripheral PD-1/PD-L1 in circulating T lymphocytes of operable breast cancer: a diagnostic test

**DOI:** 10.1186/s13000-022-01249-w

**Published:** 2022-09-10

**Authors:** Keyu Yuan, Jiangping Wu, Yanjie Zhao, Shuzhen Lyu, Quan Zhou, Feng Shi, Yanping Li, Qingkun Song

**Affiliations:** 1 Department of Breast Surgery, Beijing Shijitan Hospital, Capital Medical University, Tieyi Road 10, Haidian District, Beijing, 100038 China; 2 Department of Cancer Research, Beijing Shijitan Hospital, Capital Medical University, Tieyi Road 10, Haidian District, Beijing, 100038 China; 3 Department of Medical Oncology, Beijing Shijitan Hospital, Capital Medical University, Tieyi Road 10, Haidian District, Beijing, 100038 China; 4 Department of Pathology, Cancer Hospital, Chinese Academy of Medical Sciences, Peking Union Medical College, Panjiayuan South Lane 17, Chaoyang District, Beijing, 100021 China; 5 Department of Pathology, Beijing Shijitan Hospital, Capital Medical University, Tieyi Road 10, Haidian District, Beijing, 100038 China; 6 Department of Clinical Epidemiology, Beijing Youan, Capital Medical University, Xitoutiao 8, Fengtai District, Beijing, 100069 China

**Keywords:** Peripheral PD-1/PD-L1, Tumor microenvironment, Consistent expression, Breast cancer

## Abstract

**Background:**

The expression of PD-L1 in the immune microenvironment can guide the application of immunosuppressants. In order to monitor the immune status of the body, repeated biopsies have to be taken. Our research aims to find new and convenient means to evaluate this indicator.

**Methods:**

Eighty-three cases of newly diagnosed operable breast cancer without receiving preoperative treatment, were recruited from Beijing Shijitan Hospital between November 2018 and November 2019. The expression of PD-1/PD-L1 on circulating T lymphocytes was detected by flow cytometry and the expression of PD-L1 on immune cells in tumor microenvironment was detected by immunohistochemistry.

**Results:**

The median percentage of positive PD-1 and PD-L1 expression on circulating T lymphocytes was 15.2% and 0.7%, respectively. The peripheral PD-1 had no relationship with clinicopathological characteristics, but the peripheral PD-L1 expression had a correlation with lymph node metastasis (*p* = 0.005) and Her-2 expression (*p* = 0.034) (p < 0.05). The positive rate of PD-L1 expression was 32.9% in tumor microenvironment. PD-L1 expression in tumor microenvironment had a significant correlation with PD-1/PD-L1 expression on circulating T lymphocytes, the correlation coefficients being 0.24 (*p* < 0.05) and 0.26 (*p* < 0.05), respectively. To predict the PD-L1 expression in tumor microenvironment, the area under the receiver operating characteristic curve was 0.65 and 0.66 for peripheral PD-1 and PD-L1, respectively. High level of peripheral PD-1/PD-L1 expression was associated with the odds ratios of 5.42 and 4.76 for positive PD-L1 expression in tumor microenvironment.

**Conclusion:**

Peripheral PD-1/PD-L1 expression had a significant consistency with PD-L1 expression in tumor microenvironment and could act as an alternative choice of tissue detection, for the patients intolerable of biopsy.

## Background

With the severe challenge from breast cancer (BC) worldwide, novel therapeutic options are emerging in recent years [1–3]. The immune checkpoint inhibitors (ICIs) were approved to improve the prognosis of non-small cell lung cancer, melanoma, and many solid tumors [4, 5]. Triple-negative breast cancer (TNBC) is a particular subtype of BC, characterized by negative expression of hormone receptors and Her-2. TNBC had a high number of tumor-infiltrated lymphocytes (TILs) in tumor environment (TME) and was approved for the immunotherapy of PD-1/PD-L1 inhibitors [6]. The PD-L1 positive rate was reported to range from 38 to 78% in TNBC, and the variation was related with the ethnicity of patients, previous treatment and metastasis [7–10]. TME expression of PD-1/PD-L1 was reported to be correlated to the clinicopathological characteristics and the clinical responses to ICIs [7, 11, 12].

Immune checkpoint molecules on peripheral lymphocytes changed dramatically with treatment and reflected the clinical efficacy of anti-cancer treatment [13]. However, tissue biopsy is time-consuming, and traumatic. More than half patients were fearful and anxious to biopsy, and 5.2% of them had complications. Liquid biopsy testing in peripheral blood can avoid these limitations [14, 15], and provided an alternative choice for patients intolerable to biopsy. This study evaluated the feasibility of alternative PD-1/PD-L1 on circulating T lymphocytes to TME PD-L1 expression in BC patients.

## Methods

### Aim

The purpose of this study was to explore the correlation and consistency of PD-1/PD-L1 expression on circulating T lymphocytes and PD-L1 expression in TME.

### Ethical approval

This study was approved by the Ethics Committee of Beijing Shijitan Hospital, Capital Medical University. Patients provided the written informed consent.

### Patients

Eighty-three cases of operable BC patients were recruited at Beijing Shijitan Hospital between November 2018 and November 2019. Patients did not have any invasions to skin or chest wall. Patients did not have any diagnosis of inflammatory BC, autoimmune diseases, heart, brain, kidney, or other vital organs insufficiency. Patients should have the Eastern Cooperative Oncology Group (ECOG) > 2 and did not receive any preoperative treatments. Peripheral blood samples were collected preoperatively and tumor tissues were obtained through biopsy or surgery.

Tumor size was categorized by the diameter, ≤ 2 cm (T1), ≤ 5 cm (T2) and > 5 cm (T3). Clinical tumor node metastasis (cTNM) stage was classified as I (T_1_N_0_M_0_), II (T_0~1_N_1_M_0_, T_2_N_0~1_M0, T_3_N_0_M_0_) and III (T_0~2_N_2_M_0_, T_3_N_1~2_M_0_, T_4_N_0~3_M_0_, T_0~4_N_3_M_0_) stage. Histological grade was defined by the scores estimated according to the glandular duct formation, nuclear pleomorphism and mitotic ability. The score range of grade I II and III was 3 to 5, 6 to 7 and 8 to 9, respectively.

### Immunohistochemistry (IHC)

The positive threshold of ER and PR in IHC detection was set as 1% tumor cell staining. Positive expression of HER-2 was defined as +  +  + in IHC tests or positive in situ hybridization (ISH) test; negative expression was defined as -, + in IHC test or negative ISH test. Ki-67 index was detected by IHC on 4 μm-thick formalin fixed paraffin-embedded sections. Hot-spot area was determined under low-power field and the index ≥ 14% was defined as high expression of Ki-67. Molecular subtypes were defined as Luminal A (HER-2 negative, ER positive, Ki-67 low expression), Luminal B (HER-2 negative, ER positive, Ki-67 high expression or HER-2 positive, ER positive, Ki-67 arbitrary), TNBC (HER-2 negative, ER negative, Ki-67 arbitrary), and HER-2 overexpression (HER-2 positive, ER negative, Ki-67 arbitrary).

Monoclonal antibodies to PD-L1 (rabbit anti-human, #SP142) were purchased from Roche Shanghai Co. Ltd. Second antibodies were purchased from Beijing Zhongshanjinqiao biotechnology Co Ltd. The EnVision two-step method was used to detect the expression of PD-L1 on the immune cells in TME. Two pathologists interpreted the IHC staining of immune cells on the whole section. Tumor-infiltrating immune cells with brown staining accounting for more than 1% tumor area was determined as positive expression of PD-L1 in TME [16, 17].

### Flow cytometry

Six milliliter venous blood was collected into EDTA-K2 anticoagulation tube (Becton, Dickinson and Company) and three-color flow cytometric analysis was performed to determine cell phenotypes. The expression of PD-L1 and PD-1 on the surface of circulating T lymphocytes was detected by Cytomics FC500 flow cytometer (Beckman-Coulter). The monoclonal antibodies of CD3-FITC (A07746, Beckman-Coulter), PD-1-PE/Cy5.5 (B30634, Beckman-Coulter) and PD-L1-PE/Cy7 (A78884, Beckman-Coulter) were added into the flow tube. PD-1^+^ and PD-L1^+^ T lymphocytes was defined as percentage of total circulating T lymphocytes (CD3^+^ cells). 5000 CD3^+^ cells were gated to calculate the percentage of PD-1/PD-L1 positive T cells by a CXP analysis software (Beckman-Coulter). PD-1/PD-L1 positive T lymphocytes were gated on PD-1/PD-L1 positive cells in CD3^+^ cell gate.

### Statistical analysis

All data were analyzed by SPSS software (version 23.0). The Mann–Whitney U test was used to analyze the relationship between age, lymph node metastasis, ER, PR, HER-2, Ki-67 index and the positive levels of PD-1/PD-L1 on circulating T lymphocytes. Spearman correlation test was used to analyze the relationship between tumor size, cTNM stage, histological grade and the positive levels of PD-1/PD-L1 on circulating T lymphocytes. Kruskal–Wallis test was used to analyze the relationship between the molecular subtype and the positive levels of PD-1/PD-L1 on circulating T lymphocytes. χ2 test was used to analyze the relationship between age, lymph node metastasis, ER, PR, HER-2, Ki-67 index, molecular subtype and the TME PD-L1 expression; Mann–Whitney U test was used to analyze the relationship between the tumor size, cTNM stage, histological grade and the TME PD-L1 expression. Spearman correlation test was used to analyze the relationship between the positive levels of PD-1/PD-L1 on circulating T lymphocytes and the positive levels of TME PD-L1.

The receiver operative character (ROC) curve was illustrated between the peripheral PD-1/PD-L1 and TME PD-L1 expression, and the validity was estimated by the area under the curve (AUC). The cut-off value of peripheral PD-1/PD-L1 versus TME PD-L1 expression was calculated and the percentage of PD-1/PD-L1 on circulating T cells was transformed to categorical variable by the cut-off value. The odds ratios of expression of peripheral PD-1/PD-L1 in the categorical variables to TME PD-L1 expression were estimated in Logistical Regression Model with age adjustment. All analyses were two-tailed and significant level was 0.05.

## Results

### The positive levels of PD-1/PD-L1 in peripheral blood samples and TME

**Fig. 1:**
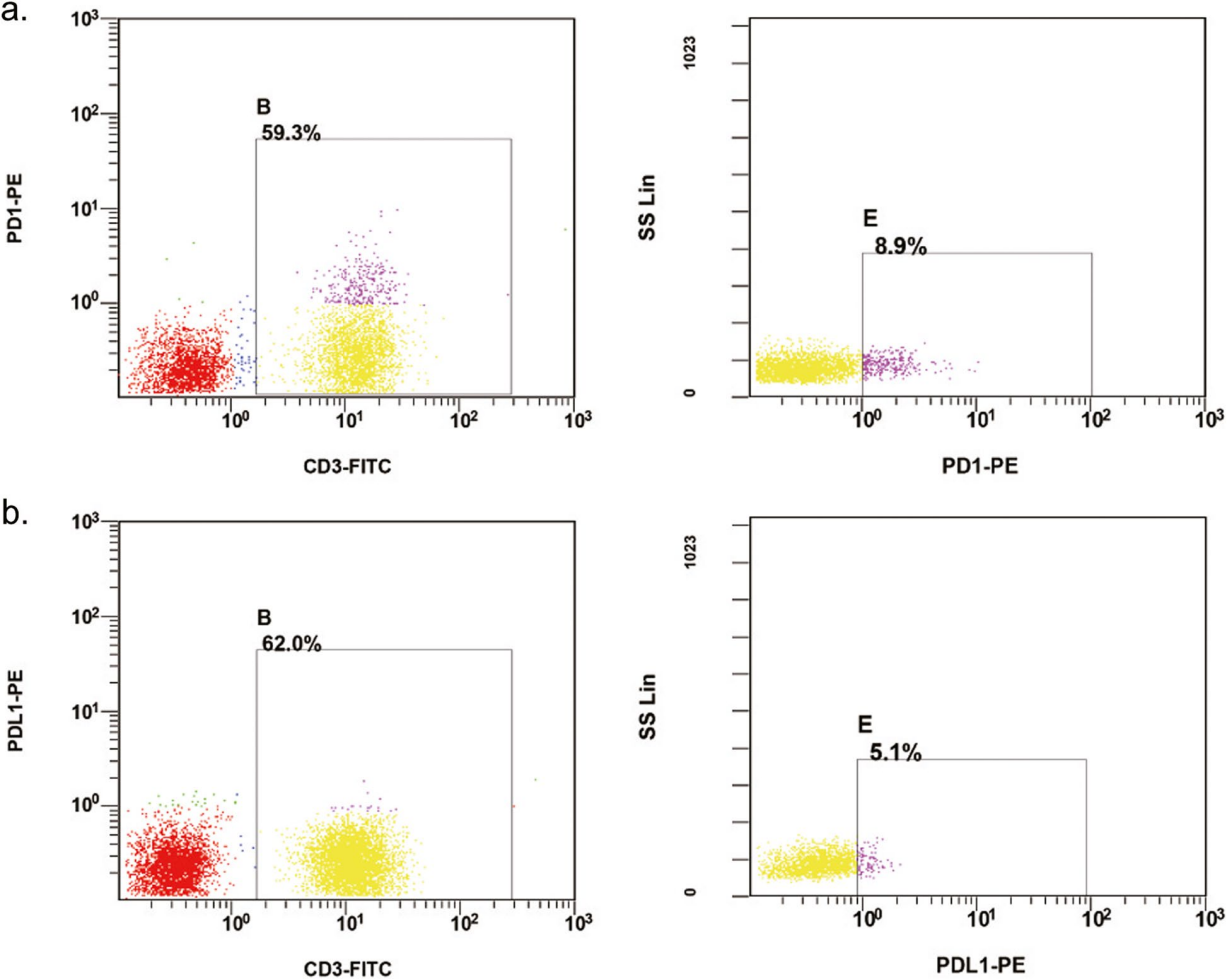
The expression of PD-1/PD-L1 on circulating T lymphocytes

**Fig. 2:**
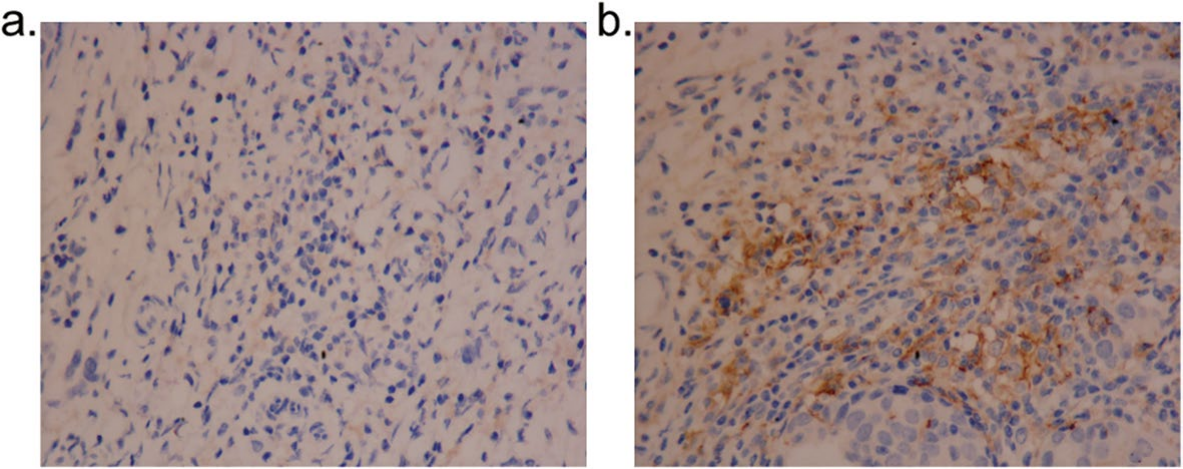
The high and the low level of PD-L1 positive immune cells in TME, **A** Patients with low level of PD-L1 positive immune cells in TME (IHC × 400), **B** Patients with high level of PD-L1 positive immune cells in TME (IHC × 400)

In peripheral blood, CD3^+^ circulating T lymphocytes had positive expression of PD-1 (Fig. 1A) and PD-L1 (Fig. 1B). The median percentage of PD-1/PD-L1 positive T lymphocytes was 15.2% and 0.7%, respectively. TME PD-L1 expression in immune cells had a heterogeneity (Fig. 2A and B) and the median percentage was 32.9%.

### Correlation with clinicopathological characteristics

The positive levels of PD-1/PD-L1 on circulating T lymphocytes did not have any correlation with age, tumor size, cTNM stage, histological grade, ER and PR status, Ki-67 index and molecular subtype (Table 1). The median percentage of PD-L1 positive circulating T lymphocytes was 1.4% in patients with lymph node metastasis, significantly higher than that in patients without lymph node metastasis (0.6%, Table 1); The median percentage of PD-L1 positive circulating T lymphocytes in HER-2 positive patients was 0.5%, significantly lower than that in HER-2 negative patients (0.9%, Table 1). The median percentage of PD-1 positive circulating T lymphocytes had no relationship with lymph node metastasis and HER-2 expression (Table 1).

**Table 1:** The relationship between the percentage of PD-1/PD-L1 positive circulating T lymphocytes and clinicopathological characters

	n	Percentage of PD-1/PD-L1 positive circulating T lymphocytes (%)			
		PD-1, median (IQR)	*P*	PD-L1, median (IQR)	*P*
Age^a^					
≤ 50	25	14.40(8.30–37.90)		0.80(0.20–3.40)	
> 50	52	15.25(3.60–41.20)	0.757	0.70(0–6.50)	0.806
Tumor size^b^					
T1	43	16.50(3.60–39.70)		0.90(0.20–6.50)	
T2	30	12.80(8.80–41.20)		0.70(0–3.40)	
T3	4	12.10(8.60–14.60)	0.227	0.55(0.40–1.10)	0.352
Lymph node metastasis^a^					
Yes	21	15.20(3.60–39.70)		1.40(0.30–3.50)	
No	55	14.60(6.80–41.20)	0.816	0.60(0–3.40)	0.005
cTNM stage^b^					
I	37	16.50(8.20–39.70)		0.60(0.20–6.50)	
II	24	17.15(3.60–41.20)		0.90(0–3.40)	
III	16	11.20(8.60–19.80)	0.066	0.55(0.10–2.50)	0.265
Histological grade^b^					
I	6	14.20(12.80–18.50)		1.15(0.10–3.20)	
II	43	16.30(3.60–28.10)		0.70(0.20–3.40)	
III	8	25.40(8.30–41.20)	0.284	1.30(0.40–3.50)	0.549
ER^a^					
Positive	54	15.25(3.60–37.90)		0.70(0.10–3.40)	
Negative	22	15.55(8.30–41.20)	0.837	0.70(0–6.50)	0.922
PR^a^					
Positive	50	15.25(3.60–41.20)		0.65(0.10–3.40)	
Negative	26	15.55(8.30–39.70)	0.996	0.75(0–6.50)	0.947
HER-2^a^					
Positive	15	12.90(8.30–30.10)		0.50(0–3.20)	
Negative	58	15.55(3.60–41.20)	0.432	0.90(0.10–3.50)	0.034
Ki-67 index^a^					
Low(≤ 14%)	22	15.10(8.20–28.10)		0.45(0.10–2.90)	
High (> 14%)	53	14.60(3.60–41.20)	0.807	0.70(0.20–6.50)	0.158
Molecular subtype^c^					
Luminal A	18	14.25(8.20–26.00)		0.70(0.10–2.90)	
Luminal B	36	16.50(3.60–37.90)		1.10(0.20–3.40)	
TNBC	13	20.79(8.60–41.20)		1.45(0.40–3.50)	
HER-2 overexpression	8	11.05(8.30–28.10)	0.450	0.40(0–6.50)	0.152

^a^Mann-Whitney U test, ^b^Spearman correlation test, ^c^Kruskal-Wallis test

The percentage of PD-L1 positive immune cells in TME had no relationship with age, tumor size, lymph node metastasis, cTNM stage, histological grade, ER/PR and HER-2, Ki-67 index and molecular subtype (Table 2).

**Table 2:** The relationship between PD-L1 expression in TME and clinicopathological characters

	PD-L1 expression		P
	Positive (*n* = 24)	Negative (*n* = 49)	
Age, n(%)^a^			
≤ 50	8(33.33%)	18(36.73%)	
> 50	16(66.67%)	31(63.27%)	0.776
Tumor size, n(%)^b^			
T1	14(58.33%)	28(57.14%)	
T2	9(37.50%)	19(38.78%)	
T3	1(4.17%)	2(4.08%)	0.930
Lymph node metastasis, n(%)^a^			
Yes	7(29.17%)	13(26.53%)	
No	17(70.83%)	36(73.47%)	0.812
cTNM stage, n(%)^b^			
I	13(54.17%)	24(48.98%)	
II	6(25.00%)	15(30.61%)	
III	5(20.83%)	10(20.41%)	0.720
Histological grade, n(%)^b^			
I	1(5.00%)	4(10.26%)	
II	14(70.00%)	31(79.49%)	
III	5(25.00%)	4(10.26%)	0.131
ER, n(%)^a^			
Positive	16(66.67%)	38(77.55%)	
Negative	8(33.33%)	11(22.45%)	0.319
PR, n(%)^a^			
Positive	15(62.50%)	33(67.35%)	
Negative	9(37.50%)	16(32.65%)	0.682
HER-2, n(%)^a^			
Positive	7(29.17%)	8(16.67%)	
Negative	17(70.83%)	40(83.33%)	0.218
Ki-67 index, n(%)^a^			
Low(≤ 14%)	6(25.00%)	15(31.25%)	
High (> 14%)	18(75.00%)	33(68.75%)	0.582
Molecular subtype, n(%)c			
Luminal A	5(20.83%)	13(26.53%)	
Luminal B	11(45.83%)	25(51.02%)	
TRIPLE-NEGATIVE BREAST CANCER	4(16.67%)	7(14.29%)	
HER-2 overexpression	4(16.67%)	4(8.16%)	0.317

^a^Chi-square test, ^b^Mann-Whitney U test, ^c^Kruskal-Wallis test

### The consistency between peripheral and TME PD-1/PD-L1 expression

The correlation coefficients between percentage of PD-1/PD-L1 positive circulating T lymphocytes and percentage of PD-L1 positive immune cells in TME were 0.24 (*p* = 0.046) and 0.26 (*p* = 0.034), respectively.

**Fig. 3:**
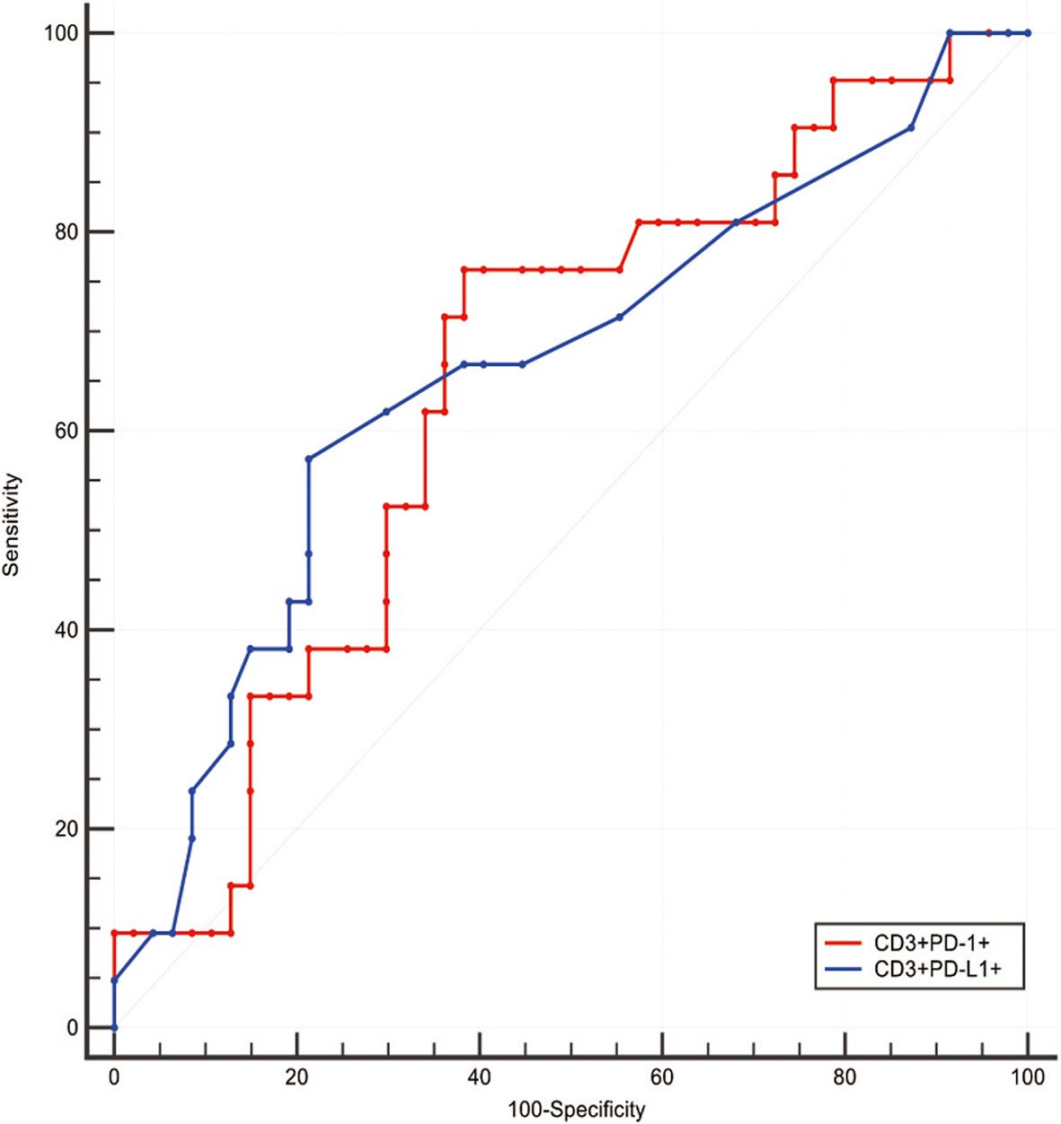
ROC curve of peripheral PD-1/PD-L1 expression and TME PD-L1 expression

The AUC between the percentage of PD-1/PD-L1 positive circulating T lymphocytes and TME PD-L1 expression was 0.65 (95%CI 0.53, 0.76) and 0.66 (95%CI 0.54, 0.77) (Fig. 3), with the cut-off values of 14.6% and 1.1%, respectively.

Higher percentage of PD-1/PD-L1 positive circulating T lymphocytes in peripheral blood was associated with a 5.42-fold (*p* = 0.007) and 4.76-fold high probability to be TME PD-L1 positive (*p* = 0.005, Table 3).

**Table 3:** The correlation between percentage of PD-1/PD-L1 positive circulating T lymphocytes and TME PD-L1 expression in Logistic Regression Model

	Crude Odds ratio (95%CI)	P	Adjusted Odds ratio^a^ (95%CI)	P
Percentage of PD-1 positive circulating T lymphocytes				
≤ 14.6%	1.00		1.00	
> 14.6%	5.16 (1.61–16.51)	0.006	5.42 (1.66–17.67)	0.005
Percentage of PD-L1 positive circulating T lymphocytes				
≤ 1.1%	1.00		1.00	
> 1.1%	4.93 (1.64–14.99)	0.005	4.76 (1.54–14.72)	0.007

^a^adjusting age

### Subgroup analysis in molecular subtypes

**Fig. 4:**
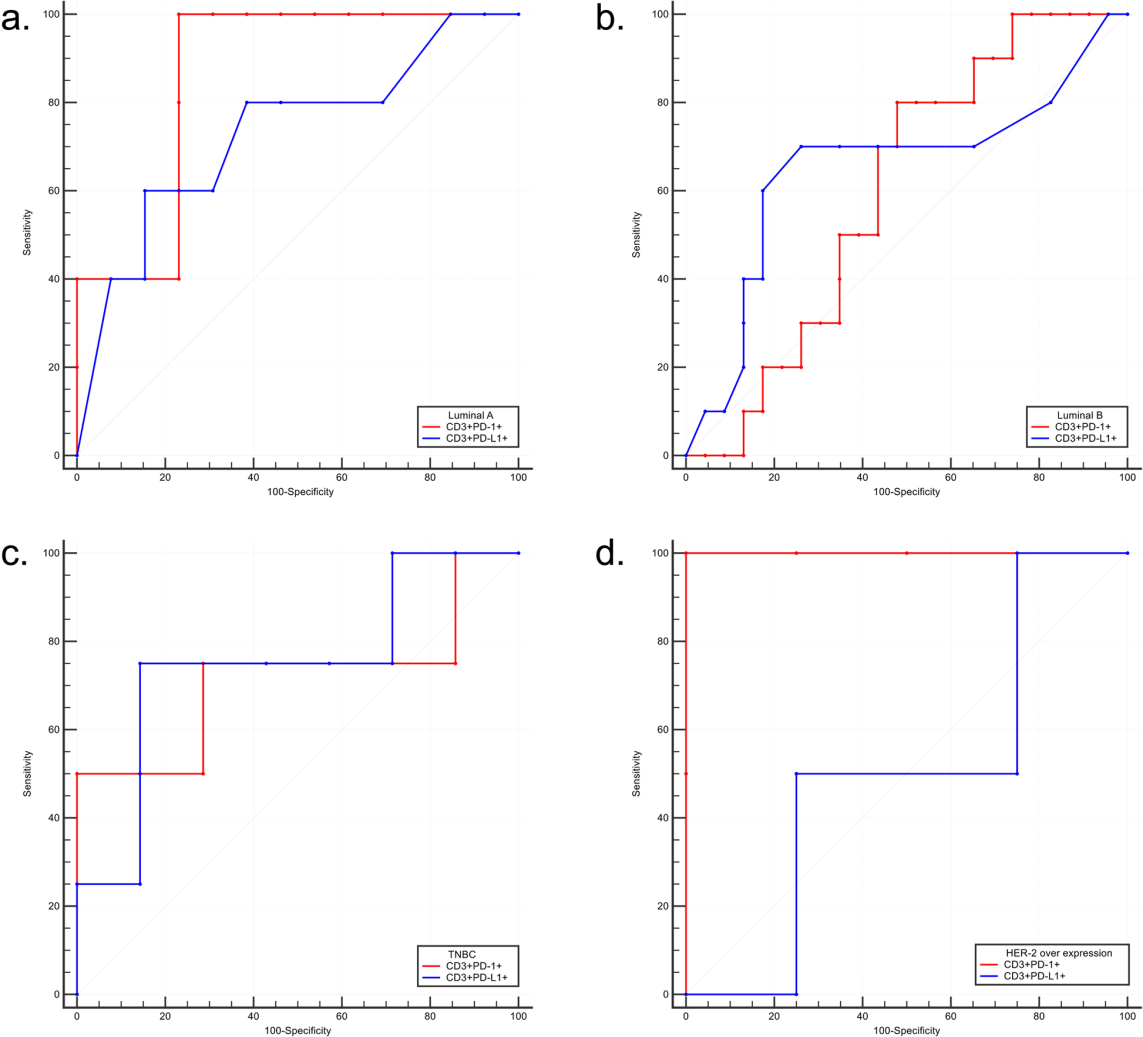
ROC curve of peripheral PD-1/PD-L1 expression and TME PD-L1 expression in molecular subtypes. **A** Luminal A, **B** Luminal B, **C** TNBC, **D** HER-2 overexpression

In Luminal A subtype, the AUC value was 0.86 (95%CI = 0.62–0.98, *p* < 0.001) and 0.73 (95%CI = 0.47–0.91, *p* = 0.12) between PD-1/PD-L1 positive circulating T lymphocytes and TME PD-L1 positive expression (Fig. 4A). In Luminal B subtype, the AUC value was 0.60 (95%CI = 0.42–0.77, *p* = 0.317) and 0.65 (95%CI = 0.47–0.81, *p* = 0.209) between PD-1/PD-L1 positive circulating T lymphocytes and TME PD-L1 positive expression (Fig. 4B). In TNBC subtype, the AUC value was 0.71 (95%CI = 0.38–0.93, *p* = 0.313) and 0.75 (95%CI = 0.41–0.95, *p* = 0.175) between PD-1/PD-L1 positive circulating T lymphocytes and TME PD-L1 positive expression (Fig. 4C). In HER-2 overexpression subtype, the AUC value was 1.00 (95%CI = 0.54–1.000, *p* < 0.001) and 0.50 (95%CI = 0.12–0.88, *p* > 0.999) between PD-1/PD-L1 positive circulating T lymphocytes and TME PD-L1 positive expression (Fig. 4D).

## Discussion

In this study, we observed a significant consistency of peripheral PD-1/PD-L1 expression with TME PD-L1 expression. BC patients with higher level of peripheral PD-1/PD-L1 were more likely to be PD-L1 positive in TME.

The Impassion 130 study revealed that PD-L1 inhibitor Atezolizumab combined with chemotherapy significantly prolonged the median PFS by 2.5 months in PD-L1 positive patients compared with chemotherapy alone [7, 8]. In our study, PD-1/PD-L1 expression on circulating T lymphocytes had a significant consistency with the PD-L1 expression in TME. Immune checkpoint proteins, including CTLA-4, CD28 and PD-1, are co-stimulatory or co-suppressive proteins expressed on the surface of antigen-presenting cells and T lymphocytes, and maintain the immune balance by up- or down-regulating T lymphocyte functions [18, 19]. Tumor cells express PD-L1 and bind to PD-1 on the immune cells to escape recognition and elimination from the immune system. The link between PD-1 and PD-L1 down-regulated the cell counts and function of T lymphocytes, and induced occurrence, progression and drug resistance of malignant cells [20, 21].

Higher level of TILs indicated a better prognosis of BC [22–24]. PD-1/PD-L1 expression in TME was the indicator for PD-1 inhibitor therapy [25, 26], and more than 1% expression rate of PD-L1 was eligible for atezolizumab treatment for BC [22, 26]. But the expression of immune checkpoint proteins in peripheral blood and TME was dynamically changing during treatment [27–30]. It is necessary to repeat tissue biopsies for the optimistic treatments. For the inconvenient tissue biopsy, liquid biopsy of peripheral blood became the alternative, and circulating T lymphocytes were reported to reflect the condition of TILs in TME [27, 31]. PD-1/PD-L1 expression on circulating T lymphocytes had a significant consistency with the TME PD-L1 expression, especially in Luminal A and HER-2 overexpression subtypes.

PD-L1 expression on circulating T lymphocytes was related to lymph node metastasis and HER-2 expression, which were not consistent with other studies [32–34]. This might be caused by the application of different antibodies and scoring standards. Since Atezolizumab was the first approved checkpoint inhibitor of PD-1/PD-L1 for BC immunotherapy, we chose the recommended antibody SP142 and the results are more reliable [26, 35]. Previous treatments and clinical stage affected PD-1/PD-L1 expression and immunotherapy efficacy [36–40]. The recruited patients were in early or mid-stage and never received any treatment preoperatively. Heterogeneity of research subjects between studies contributed to the variation in results. The positive rate of TME PD-L1 was 36%, in consistent with the Impassion130 trial in Japan [9].

The limited sample size was the first limitation in this study. The low percentage of PD-L1 positive circulating T lymphocytes was the second limitation. Lack of soluble PD-1/PD-L1 detection in peripheral blood was another limitation.

## Conclusions

Peripheral PD-1/PD-L1 expression on circulating T lymphocytes had a certain correlation and consistency with TME PD-L1 expression, and was potential to be an alternative to the TME detection, especially for the patients intolerable to tissue biopsy. However, this result still needs to be verified with a larger sample size and we will definitely continue to conduct subsequent studies.

## Data Availability

The datasets generated and analysed during the current study are not publicly available due to privacy and ethical but are available from the corresponding author on reasonable request.

## Abbreviations

AUC: Area under the curve
BC: Breast cancer
cTNM: Clinical tumor node metastasis
ECOG: Eastern Cooperative Oncology Group
ER: Estrogen Estrogen
HER-2: Human Epidermal Growth Factor Receptor Type 2
ICI: Immune checkpoint inhibitor
IHC: Immunohistochemistry
ISH: In situ hybridization
PD-1: Programmed Death-1
PD-L1: Programmed Death Ligand-1
PR: Progesterone Estrogen
ROC: Receiver operative character
TIL: Tumor-infiltrated lymphocyte
TME: Tumor environment
TNBC: Triple-negative breast cancer
